# Context-Dependent Sensitivity to Losses: Range and Skew Manipulations

**DOI:** 10.1037/xlm0000629

**Published:** 2018-10-25

**Authors:** Lukasz Walasek, Neil Stewart

**Affiliations:** 1 Department of Psychology, University of Warwick; 2 Warwick Business School, University of Warwick

**Keywords:** loss aversion, decision by sampling, context, range effect, skew effect

## Abstract

The assumption that losses loom larger than gains is widely used to explain many behavioral phenomena in judgment and decision-making. It is also generally accepted that loss aversion is a stable, traitlike individual difference characterizing people’s sensitivity to gains and losses. This interpretation was recently challenged by Walasek and Stewart (2015), who showed that by manipulating the range of the gains and losses used in the accept−reject task it is possible to find loss aversion, loss neutrality, and a reversal of loss aversion. Here, we reexamined the claim that these context effects arise as a result of people being sensitive to the rank position of a given gain among other gains and the rank position of a loss among other losses. We used skewed distributions of outcomes to manipulate the rank position of gains and losses while keeping the range of possible outcomes constant. We found a small but robust effect of skew on the propensity to accept mixed gambles. We compared the sizes of skew and range effects and found that they are of similar magnitude but that the range effects are smaller than those reported by Walasek and Stewart. We were able to attenuate loss aversion, but we were not able to replicate Walasek and Stewart’s reversal of loss aversion. We conclude that rank effects are, at least in part, responsible for the loss aversion seen in the accept−reject task.

Under the loss aversion hypothesis, the hedonic impact of losses is considerably higher than the hedonic impact of equivalent gains. The assumption that losses loom larger than gains remains one of the more important theoretical contributions in behavioral sciences, used to explain people’s choices and valuations in both risky and riskless contexts (Camerer, 2005). Loss aversion is also an important component of the prospect theory (Kahneman & Tversky, 1979) and was included in the model to explain why people tend to reject a fair offering of a 50/50 chance to win $X or lose $X. Early experiments on risky choice estimated the value of loss aversion at λ = 2.25, which reflects the widespread belief that the disutility of a loss is just over twice as high as the utility of an equivalent gain (Tversky & Kahneman, 1992).

Many existing models of choice behavior incorporate loss aversion as a primitive that represents a hardwired feature of people’s preferences (e.g., Kőszegi & Rabin, 2006; Usher & McClelland, 2004). Indeed, it has been shown that various biological systems might underlie the gain−loss asymmetry. Existing work has found that people’s overweighting of losses is reflected in their genotype (Frydman, Camerer, Bossaerts, & Rangel, 2011; Zhong, Chark, Ebstein, & Chew, 2012) and hormone production (Chumbley et al., 2014) and directly in activation of brain regions related to processing of emotions (Chib, De Martino, Shimojo, & O’Doherty, 2012; De Martino, Camerer, & Adolphs, 2010; Sokol-Hessner, Camerer, & Phelps, 2013) and rewards (Tom, Fox, Trepel, & Poldrack, 2007). The assumption of traitlike loss aversion is also prevalent in studies investigating how the gain−loss asymmetry varies as a function of age (Wolf, Wright, Kilford, Dolan, & Blakemore, 2013), ethnography (Tanaka, Camerer, & Nguyen, 2010; Tanaka & Munro, 2014), or education (Hjorth & Fosgerau, 2011).

There are some challenges to the notion that loss aversion is a stable individual characteristic of an individual decision maker. A growing number of studies have found no evidence of behavioral loss aversion in risky choice. Yechiam and Hochman (2013b) reported that out of 11 studies in which the effect of losses was examined using symmetric lotteries, only four found evidence of loss aversion. In studies where probabilities and outcomes were not explicitly provided to the participants but had to be learned through repeated sampling (i.e., the decision from experience paradigm; Hertwig, Barron, Weber, & Erev, 2004), no evidence of loss aversion was found in 13 unique studies. Additionally, behavior inconsistent with loss aversion was observed in experiments that used small monetary payoffs or when the safe options were framed as the status quo (see Ert & Erev, 2011, for a review). Taken together, the fact that loss aversion is highly dependent on the features of the elicitation procedure suggests that overweighting of losses in risky choice may not be a stable property of people’s preferences.

In an attempt to explain why estimates of loss aversion seem so malleable, Walasek and Stewart (2015) tested whether some of the context dependency of loss aversion could be explained by the decision by sampling (DbS) model (Noguchi & Stewart, 2014; Stewart, Chater, & Brown, 2006; Stewart, Reimers, & Harris, 2015; Stewart & Simpson, 2008). In this framework, choices are driven by accumulating the outcomes of multiple comparisons between the attributes of the alternatives on offer. The alternative chosen is the one whose attributes win the most comparisons. Because the comparisons are ordinal, what matters is the rank position of an attribute value within the set of attributes being compared. The rank position determines the fraction of comparisons that will favor the target attribute. The worst ranking attribute will not win any comparisons. The best ranking attribute will win all of the comparisons. Next we explain how DbS predicted loss aversion and its elimination and reversal in advance for a series of experiments run by Walasek and Stewart (2015) and revisit some of their claims about whether DbS offers an accurate account of the origins of loss aversion.

In four experiments, Walasek and Stewart (2015) presented their participants with a series of mixed 50/50 gambles. For example, would you accept or reject the opportunity to play a 50/50 gamble where you can gain $10 or lose $5? A loss-averse person with a λ = 2 would be indifferent concerning whether to accept or reject this gamble. A λ of 2 means that losses are weighted twice as heavily as gains, so a loss of $5 matches a gain of $10 in subjective value. In Walasek and Stewart’s experiments, the range of the distributions of gains and losses in the series of gambles varied between the experimental conditions. Consider the two asymmetric ranges of gains (Gs) and losses (Ls) presented in the left panel of Figure 1. In the G_Wide_ − L_Narrow_ condition (left panel, top) the range of gains is wider ($0−$40) than the range of losses ($0−$20), whereas the opposite is true in the G_Narrow_ − L_Wide_ condition, with a narrower range of losses ($0−$20) and wider range of gains ($0−$40; left panel, bottom). This means that any given sum of money will have a different position in the range of gains compared to the range of losses. For example, consider a gamble that occurs in both conditions and offers 50% chance of winning $17 and 50% chance of losing $17 (see the dashed lines). In the G_Wide_ − L_Narrow_ condition, according to the DbS, this gamble will be less attractive and therefore less likely to be accepted. In this condition, the gain of $17 ranks as fifth out of 10 among other gains, whereas the $17 loss ranks as ninth out of 10 among other losses. A midranking gain paired with one of the very worst losses does not seem very attractive. These ranks are reversed in the G_Narrow_ − L_Wide_ condition, where the same gamble should be much more likely to be accepted. A midranking loss paired with one of the very best gains seems very attractive. Thus one finds the prediction of loss aversion in the G_Wide_ − L_Narrow_ condition and the reverse of loss aversion in the G_Narrow_ − L_Wide_ condition according to DbS. The results presented by Walasek and Stewart were consistent with these predictions, showing a dramatic change in observed loss aversion between the conditions. When the range of gains exceeded the range of losses, the aggregate loss aversion coefficient was about 2, as is often observed. But when the range of losses exceeded the range of gains, the aggregate loss aversion coefficient was lower than 1, showing the expected reversal of loss-averse behavior.[Fig-anchor fig1]

Despite observing strong context effects in loss aversion, the design used by Walasek and Stewart (2015) does not isolate rank position as the cause. Because the distribution of gains and losses was always uniform, the rank position and position within the range were confounded. One might expect range and rank to play separate roles, an idea instantiated in the range frequency theory of the evaluation of magnitudes (Parducci, 1965). In the present work, we set out to determine whether people are truly sensitive to the rank position of a gain among other gains and a rank position of a loss among other losses. This approach allowed us to determine whether DbS offers an accurate model of context-dependent loss aversion.

In the following three experiments, we manipulated the skewness of distributions of gains and losses while controlling (i.e., keeping constant) their range. If people respond to the rank position of gains and losses, then this manipulation should influence the magnitude of loss aversion in a manner consistent with DbS. This design is illustrated in the right panel of Figure 1. Consider common values of gaining and losing $16 (see the dashed lines). In the condition with negatively skewed distribution of gains and a positively skewed distribution of losses (G_Negative (Neg)_ – L_Positive (Pos)_; middle line) $16 ranks fifth out of seven within the losses but only third out of seven within the gains, which should lead to the gamble being rejected. But in the condition with a positively skewed gains and negatively skewed losses (G_Pos_ – L_Neg_; bottom line) the ranks are reversed, which should lead to the gamble’s being accepted. Rates of acceptance should fall in between these two conditions, where gains and losses are uniformly distributed (G_Uniform(Uni)_ – L_Uni_; top line), where the gain of $16 and the loss of $16 both rank fourth out of seven in their respective distributions. We tested these predictions in the following three experiments.

## Experiment 1

### Method

#### Design

**Figure 1 fig1:**
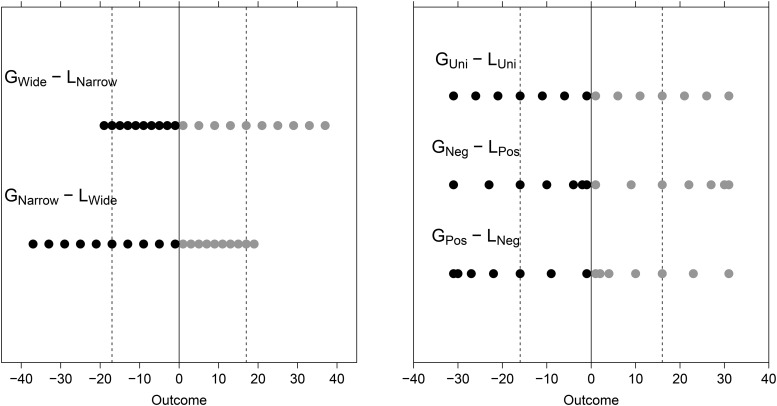
Left panel: Example stimuli with asymmetric uniform distributions of gains and losses (the manipulation used by Walasek & Stewart, 2015). At the top, gains span a wider ranger than do losses (G_Wide_ – L_Narrow_). At the bottom, losses span a wider range than do gains (G_Narrow_ – L_Wide_). The dashed lines highlight values that are common in the two conditions (−$17 or +$17). Right panel: The distributions of gains and losses used in this article. The top row shows symmetrical, uniform distributions of gains and losses (G_Uni_ – L_Uni_). The middle row shows a positively skewed distribution of losses and a negatively skewed distribution of gains (G_Neg_ – L_Pos_). This asymmetry is reversed in the bottom row (G_Pos_ – L_Neg_). Dashed lines indicate a common value shared across distributions (−$16 or +$16). G = gain; L = loss; Uni = uniform; Neg = negative; Pos = positive.

Participants were randomly allocated to one of three conditions: G_Pos_ – L_Neg_; G_Neg_ – L_Pos_; or G_Uni_ – L_Uni_. Figure 1 displays all gains and losses (but here in British pound sterling) that were used to construct 49 unique gambles for each condition (see the table in Appendix A for a list of the outcomes used). The participants’ task was to simply indicate whether they would be willing to accept and play each gamble, answering with buttons marked *Strongly Reject, Weakly Reject, Weakly Accept, and Strongly Accept*.

According to DbS, the subjective value of gambles arises from ordinal comparisons within a person’s memory. We therefore included a memory test to determine whether our participants could remember gains and losses to which they were exposed during the lottery task. In the two conditions where the skew of the distribution was manipulated, the list included 20 gains and losses that were present in the gamble task, as well as 22 that either did not occur or occurred but with a different sign (e.g., −£3 [US$4.02] present, £3 not present). In the condition where the same uniform distributions were used for gains and losses, 28 of the presented values did in fact occur in the gamble task, and 14 did not. This unavoidable asymmetry in task design allowed us to make meaningful comparison between only conditions with skewed distributions.

#### Participants

We recruited 275 participants from the crowdsourcing platform Prolific Academic (https://prolificacademic.co.uk/) in exchange for £1.00 (US$1.34). We chose the sample size to be comparable to the one used by Walasek and Stewart (2015), who found large effects with cell sizes of about 100 participants. All experiments were approved by the institutional research ethics committee.

#### Procedure

**Figure 2 fig2:**
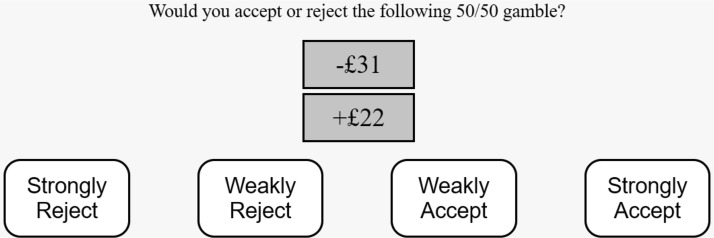
Screenshot of an accept−reject trial from Experiments 1 and 2.

Participants were informed that they would be presented with a series of lotteries and required to evaluate whether they would like to play the lottery or not. They were also shown an example of a lottery offering a 50% chance of gaining £31 (US$41.54; maximum gain in all conditions) and losing £31 (maximum loss in all conditions). The payouts mechanism was explained using a coin-tossing analogy. Participants were then presented with 49 lotteries, one after the other, in a different random order for each participant. They were given unlimited time to accept or reject them. Figure 2 displays a screenshot of an accept−reject trial.[Fig-anchor fig2]

Next, in the memory task, participants were presented with individual gains and losses and required to indicate whether they had featured in the lottery task by pressing *Yes* and *No* buttons. The left and right position of these buttons was counterbalanced across participants.

#### Modeling approach

We used the commonly used version of the prospect theory, in which the value function is parameterized as

$$\begin{array}{l} v\left(x\right)=\;x^{\alpha },\;if\;x\geq \;0\\ v\left(x\right)=\;-\text{λ}{\left|x\right|}^{\beta },\;if\;x<0 \end{array}$$ 1

where λ controls the relative slope of the gain−loss portions of the value function, α controls the curvature of the gains part of the value function, and β controls the curvature of the loss part of the value function. Following the recommendation made by Nilsson, Rieskamp, and Wagenmakers (2011), we set α = β to improve the recoverability of λ (see also Stewart, Scheibehenne, & Pachur, 2018).

We then used the exponentiated Luce’s choice rule, according to which the probability of accepting a mixed gamble is given by

$$P\left(accept\right)=\frac{e^{bias}e^{\left(w\left(\frac{1}{2}\right)gain^{\alpha }\right)}}{e^{bias}e^{\left(w\left(\frac{1}{2}\right)gain^{\alpha }\right)}+e^{\left(\;w\left(\frac{1}{2}\right)\text{λ}\;loss^{\text{β}}\right)}}=\frac{1}{1+e^{-\left(bias+w\left(\frac{1}{2}\right)gain^{\alpha }-\;w\left(\frac{1}{2}\right)\text{λ}\;loss^{\text{β}}\right)}}$$ 2

Here, *w*(1/2)*gain*^α^ is the subjective value of the gain part of the gamble and, as such, is the evidence for accepting the gamble. On the other hand, −*w*(1/2)λ*loss*^β^ is the subjective value of the loss part of the gambles and, as such, is the evidence for rejecting the gamble. The *bias* parameter simply controls for the participants’ propensity to accept gambles regardless of their outcomes (Stewart et al., 2015). We used the Nelder-Mead algorithm to estimate maximum likelihood values for α, λ, and *w*(1/2). For all parameters, we report condition median values along with their bootstrapped 95% confidence intervals (CIs). We also report results from fitting a more complex version of the prospect theory, in which α and β are allowed to differ, in the table in Appendix B.

All responses were recoded into *accept* or *reject* categories. We decided in advance to exclude the 5% of participants with the poorest model fit based on Nagelkerke’s *R*^2^ (see also Walasek & Stewart, 2015), with the objective of removing participants who responded most randomly and inconsistently.

### Results and Discussion

**Figure 3 fig3:**
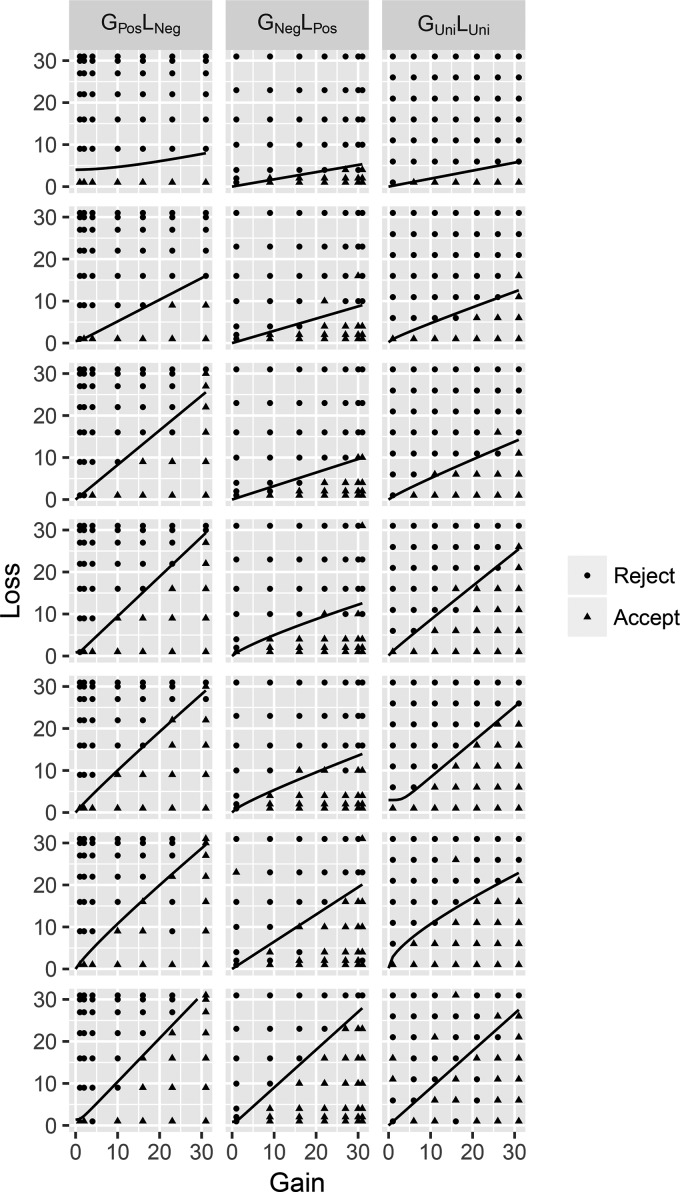
Examples of model fits to 21 randomly selected individual data from Experiment 1. Each column represents a condition, showing seven participants ordered vertically by the area under the curve. The black lines depict the best fitting indifference curves for each individual. G = gain; L = loss; Pos = positive; Neg = negative; Uni = uniform.

Figure 3 shows raw data from a random sample of participants (seven per condition). Each point in the two-dimensional gain−loss space corresponds to one gamble. Quite sensibly, participants tended to accept gambles in the southeast corner of the space, with high gains and low losses, and reject gambles in the northwest corner of the space, with low gains and high losses. By setting *P*(*Accept*) = 1/2 in Equation 2, we could derive the curve upon which people will be indifferent concerning accepting and rejecting:

$$loss=\;{\left(\left[\frac{bias}{w\left(\frac{1}{2}\right)}+gain^{\text{α}}\right]\right)}^{\frac{1}{\text{β}}}$$ 3

[Fig-anchor fig3]

The black lines in Figure 3 are the best fitting indifference curves. It is clear that the model provides an exceptionally good fit to the data, with all lines visibly separating the accept and reject regions.

In Table 1 we list aggregate parameter values along with their bootstrapped 95% confidence intervals. We found no difference in the loss aversion λ but found that participants in the G_Pos_ – L_Neg_ condition were more risk-averse than in the G_Neg_ – L_Pos_ condition (median α difference = .683, 95% CI [.108, 1.312]). However, results are sensitive to the model specification. In the table in Appendix B, we report all parameter estimates with the addition of the β parameter. Here, we no longer observed any effect on α but instead found a difference in the *bias* parameter, with people’s being much more likely to accept gains in the G_Pos_ – L_Neg_ condition than in the G_Neg_ – L_Pos_ condition (median *bias* difference = .759, 95% CI [.287, 1.311]).[Table-anchor tbl1]

Although the two models we chose are commonly used in the literature, we argue that both versions of the prospect theory are simply too complex for the type of data that we obtained for each participant in the accept−reject task. The parameters could trade off against one another. Others have argued that it is difficult, if not entirely impossible, to estimate all three parameters (α, β, λ) simultaneously from choice data because of the parameter trade-offs (see also Nilsson et al., 2011; Pachur & Kellen, 2013; Stewart et al., 2018). Indeed, we observed strong parameter correlations in log space (see the figure in Appendix C). Over and above these issues, in our own work (Walasek & Stewart, 2018; see also Spektor & Kellen, 2018), we recently showed that λ cannot be reliably recovered from responses from the (frequently used) accept−reject task.

To avoid problems of parameter recoverability under different model specifications, we took a different approach to quantifying the behavioral effect of skew manipulation. We used the area under the indifference curve, which is the fraction of gain−loss space in which participants are more likely than not to accept gambles. Walasek and Stewart (2018) showed that even when the indifference curve is governed by all of the estimated parameters, and even if these parameters trade off against one other, the area under the curve (AUC) can be reliably estimated and is an unbiased estimate of the overall propensity to accept and reject mixed gambles in the accept−reject task. In short, the AUC is the fraction of the gain−loss space in which people accept and can therefore be interpreted as the magnitude of loss aversion.

**Figure 4 fig4:**
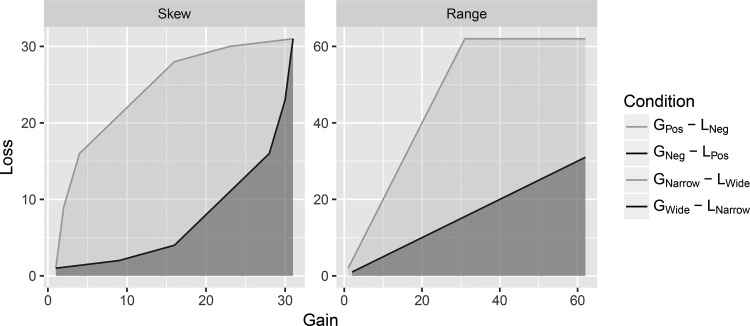
Decision by sampling predictions for differences in area under the curve in groups where the skew (left panel) and range (right panel) of gains and losses were manipulated. G = gain; L = loss; Pos = positive; Neg = negative.

Under the assumption that participants’ working memory contains information from only the immediate context (i.e., the experimental setting), DbS makes clear predictions about the AUC and the difference in AUCs between the skew conditions. For the two asymmetric conditions, G_Pos_ – L_Neg_ and G_Neg_ – L_Pos_, these are plotted in the left panel of Figure 4. The DbS indifference curves simply join gambles where the rank position of the gain among gains is equal to the rank position of the loss among losses. DbS predicts that the difference in AUCs between the G_Pos_ – L_Neg_ condition and the G_Neg_ – L_Pos_ condition will be .544. Visually, in Figure 4 the .544 is represented by the difference between the light grey and the dark grey areas.[Fig-anchor fig4]

**Figure 5 fig5:**
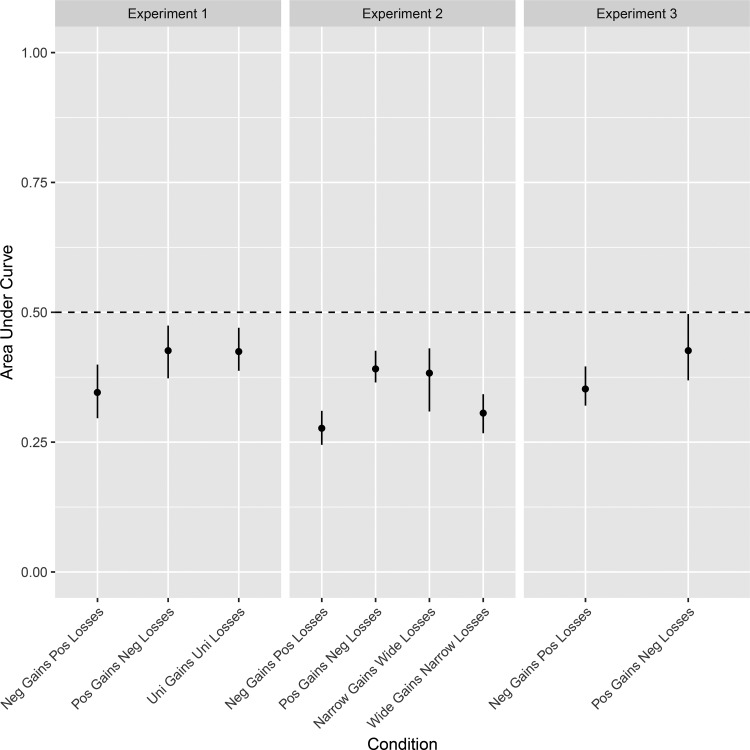
Condition median area under the curve scores (dots) for all three experiments. Error bars represent 95% bootstrapped confidence intervals around the group medians. The dashed line represents loss neutrality, with loss averse responding below the line and the opposite above the line. Neg = negative; Pos = positive; Uni = uniform.

To compute the AUC from prospect theory parameter values, we simply integrated the function in Equation 3. Aggregate values of the AUC based on the four-parameter version of the prospect theory are listed in the rightmost column of Table 1 and are also plotted in the left panel of Figure 5. The table in Appendix B also lists AUC scores obtained from the five-parameter version of the prospect theory.[Fig-anchor fig5]

It is clear that the direction of the effect is consistent with the predictions of DbS. However, the difference in AUCs between the G_Pos_ – L_Neg_ condition and the G_Neg_ – L_Pos_ condition is .079 (95% CI [.018, .143]) for the more complex version of the model and .080 (95% CI [.005, .149]) for the simpler version. The effect is smaller than predicted by the DbS. We return to the size of the effect predicted by DbS in the General Discussion.

Finally, we computed memory scores for all participants, summing the number of times they correctly recognized a gain or a loss in a memory task. We correlated memory performance with AUC scores in all three conditions. The correlations were weak in all groups: *r*(87) = −.039, 95% CI [−.245, .171], in the G_Pos_ – L_Neg_ group; *r*(86) = −.024, 95% CI [−.232, .187], in the G_Neg_ – L_Pos_ group; and *r*(82) = −.036, 95% CI [−.248, .748], in the G_Uni_ – L_Uni_ group. Thus, memory of gains and losses was only weakly associated with the tendency to accept mixed gambles.

Overall, we found no evidence of context effects induced by the skew manipulation when we measured loss aversion in terms of the prospect theory’s parameters α, β, and λ because these parameters cannot be reliably recovered from the accept−reject task. Using an alternative measure that captures the overall propensity to accept or reject gambles, we found a small effect of the skew of the distributions of gains and losses on loss aversion.

## Experiment 2

The objective of Experiment 2 was to replicate the results of Experiment 1 with a new sample of participants. Additionally, we also included manipulations of the range of gains and losses in an effort to replicate the findings of Walasek and Stewart (2015). By including manipulations of range and skew in the same experiment we could compare their effect sizes using the same model and the same measurements.

### Method

#### Design

The two skew conditions were identical to those in Experiment 1. We also introduced two conditions with range manipulation: G_Wide_ – L_Narrow_ and G_Narrow_ – L_Wide_. The table in Appendix A lists values used to form these distributions. The two range manipulations mimic the design used by Walasek and Stewart (2015; see also Tom et al., 2007). As predicted by the DbS model and described in the introduction, we expected that when the range of gains is larger than the range of losses (G_Wide_ – L_Narrow_), people will be loss averse (i.e., λ > 1). When the asymmetry is reversed (G_Narrow_ – L_Wide_), one should observe the reversal of loss aversion (i.e., λ < 1).

#### Participants

A total of 405 participants on Prolific Academic completed the experiment in exchange for £1.00. We chose the sample size so that we would have at least 100 participants per condition. Participants were randomly allocated to one of the four conditions.

#### Procedure

Participants were presented with the instructions and an example lottery in exactly the same way as in Experiment 1. We did not include the memory task from Experiment 1.

### Results and Discussion

We used the same modeling approach as in Experiment 1 and removed the top 5% of poorest fits, as planned in advance. Median parameter values are listed in Table 1. Consistent with the results of Experiment 1, we found no differences in median λ between the G_Pos_ – L_Neg_ and G_Neg_ – L_Pos_ conditions. Also consistent with Experiment 1, we did observe a significant difference in the α parameter between the two asymmetric skew conditions. In the case of more complex versions of the prospect theory, we found no differences in α, β, λ and *bias* (see the table in Appendix B).

Using the AUC measure, we found small effects, such that participants in the G_Pos_ – L_Neg_ condition were willing to accept more mixed gambles than were participants in the G_Neg_ – L_Pos_ condition. The difference in AUCs between the skew conditions was .115 (95% CI [.073, .162]) and .103 (95% CI [.062, .160]) for the model with different α and β parameters. This effect is visible in the middle panel of Figure 5, where the confidence intervals of the two treatment groups do not overlap. As in Experiment 1, the direction of the difference in loss aversion is consistent with the DbS predictions. However, both conditions show an overall level of loss aversion, and we return to this issue in the General Discussion.

We now turn to the analysis of the conditions with range manipulation. Here, we used the same model as before (see Equation 2) to estimate parameters of the prospect theory. Our results depart considerably from both predictions of the DbS and the results reported by Walasek and Stewart (2015). Here, we found that people are slightly loss-averse in both the G_Wide_ – L_Narrow_ (λ = 1.583, 95% CI [1.428, 1.735]) and the G_Narrow_ – L_Wide_ (λ = 1.344, 95% CI [1.064, 1.587]) conditions. The difference is small, and confidence intervals encompass 0: difference in λ −.239 (95% CI [−.559, .047]). This is also true for the model with α and β free to vary, in which the difference in λ −.064 (95% CI [−.257, .153]). The discrepancy between our findings and those reported by Walasek and Stewart is most likely due to the parameter trade-off discussed earlier.

We also used the AUC measure for the range manipulation. DbS predictions for the two conditions with asymmetric ranges are shown in the right panel of Figure 4. Here, the model predicts a difference in the AUC of exactly .5. By our design, DbS predicts almost the same size effect in terms of the AUC for the range and skew manipulations used in our experiments. Using the AUC, we found a small difference between the groups, with asymmetric ranges of gains and losses of .077 (95% CI [−.007, .137]) and .093 (95% CI [.006, .167] in the more complex model. Thus, the difference is similar in magnitude to that of the skew in Experiments 1 (.08) and 2 (.115).

Taken together, we found that neither range nor skew manipulations exert strong effect on people’s preferences. We found weak and similar-sized effects of skew and range on people’s overall tendency to accept and reject mixed lotteries.

## Experiment 3

In Experiment 2, we were able to replicate the small effect of skew manipulation on the probability to accept mixed gambles observed in Experiments 1 and 2. In Experiment 3, we explored whether the small effect of skew on the displayed level of loss aversion could be due to the low salience of the skews in the distributions of experienced gains and losses. To enhance our skew manipulation, in Experiment 3 we made the distributions of gains and losses visible to our participants. This is a heavy-handed manipulation, and if the effect of skew remained small we could be confident it was indeed a relatively small effect.

### Method

#### Design

**Figure 6 fig6:**
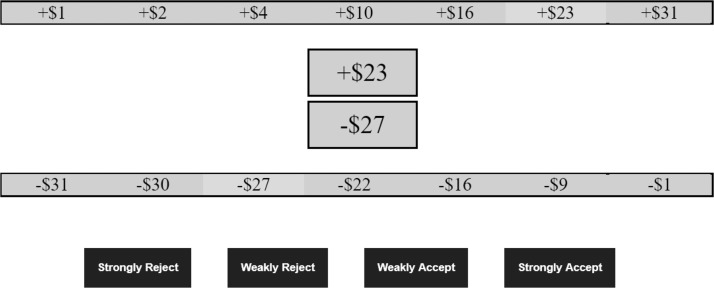
Screenshot of an accept−reject trial from Experiment 3.

Participants were randomly allocated to one of two conditions, either the G_Pos_ – L_Neg_ or G_Neg_ – L_Pos_ condition. The possible monetary values in these conditions were the same as in previous experiments (see the table in Appendix A) with the exception that all outcomes were in U.S. dollars. We made one change to the display format of the lottery task. On each trial, participants saw a lottery together with two-strip displays of possible gains and losses. Amounts on offer for a given lottery were highlighted as seen in Figure 6, with the intention of making rank position particularly salient.[Fig-anchor fig6]

#### Participants

This time, 188 participants from the crowdsourcing platform Amazon Mechanical Turk completed the experiment in exchange for $1.00. We aimed for approximately 90 participants per condition.

#### Procedure

Participants were presented with the instructions and an example lottery in exactly the same way as in Experiments 1 and 2. The only exception was that the instructions included the display of the possible distributions of gains and losses. Participants were also informed that these values were all possible outcomes and that on each trial a gain and a loss would be drawn at random from these sets.

### Results and Discussion

Following our exclusion criteria, as we planned in advance, we removed the top 5% of highest deviance scores (10 participants). Median parameters of our model fitting are listed in Table 1, and median AUC scores are plotted in Figure 5. The results closely replicate the findings in Experiments 1 and 2, showing a larger AUC (i.e., less loss aversion) in the G_Pos_ – L_Neg_ condition than in the G_Neg_ – L_Pos_ condition. The difference in AUCs between the conditions was small, with a median difference of .074 (95% CI [.002, .149]) and .068 (95% CI [−.005, .140]) in the more complex version of the prospect theory.

## General Discussion

In three experiments, we reexamined the claim that the distribution of gains and losses used in the elicitation procedure influences how likely people are to accept 50/50 gambles for simple gains and losses. More specifically, we manipulated the skew of the distribution of gains and losses to test whether DbS offers an accurate model of the origin of loss aversion. According to DbS, the subjective value of a mixed gamble is based on the rank position of the gain among other gains and the loss among other losses. We found that people are more likely to choose to play a given gain−loss pair gamble when gains are positively skewed and losses are negatively skewed. This is the prediction that DbS makes: A given gain looks better when most of the other gains being offered are smaller (as in a positively skewed distribution) and a given loss looks better when most of the other losses being offered are larger (as in a negatively skewed distribution). We replicated this finding across three experiments. These effects of skew lead us to conclude that rank effects are responsible, at least in part, for the loss aversion seen in the accept−reject task.

We found difficulty in estimating prospect theory parameters from the accept−reject task data. Recently, this model recovery problem has been identified and is now well understood (Nilsson et al., 2011; Pachur & Kellen, 2013; Spektor & Kellen, 2018; Walasek & Stewart, 2018). What this means is that we could not draw conclusions about how prospect theory parameters change with range and skew manipulations in the accept−reject task, because prospect theory parameters cannot be reliably identified using accept−reject task data. Our solution was to use a more robust area-under-the-curve (AUC) measure, which captures the fraction of gain−loss space in which people are more likely than not to accept the gamble.

We also compared the size of the effects of manipulating skew with the size of the effects of manipulating range. Using the AUC measure, we found similar-sized effects. The effects of range and skew were reasonably small, and they were smaller than the effects that DbS predicts under the assumption that the only gains and losses in memory are those from the experiment. Why was the observed effect of skew smaller than expected? In DbS, comparisons between attribute values can involve intraexperiment comparisons (as in Stewart et al., 2015) but also comparisons to distributions of attribute values from outside the experimental context (as in Stewart et al., 2006). Given our experimental effects are small compared to the pure within-experiment comparison-only predictions (see Figure 4), it could be that comparisons to a set of attribute values from outside the experiment play a significant role. Without knowing what the distribution of extraexperiment attribute values in memory is, DbS makes predictions for only the *direction of the difference* between conditions (see Stewart et al., 2015, pp. 692–693). For the small effects of skew and range to be consistent with DbS, we would have had to assume that the effect of intraexperiment gains and losses had been diluted by the (unmeasured) extraexperiment gains and losses.

We also found, in Experiment 1, that people’s memory of gains and losses does not correlate strongly with sensitivity to losses. DbS predicts that context effects should be stronger in people who show a better memory for the context. To enhance people’s awareness of the skewed distributions, we explicitly showed them to participants in Experiment 3. This, however, did not enhance the overall size of the effect of skew on the probability of accepting lotteries.

We also reanalyzed data from all four experiments reported by Walasek and Stewart (2015) using the new AUC measure. First we comment on the difference between the conditions and then comment on whether we observed a reversal of loss aversion in the G_Narrow_ – L_Wide_ condition. As we outlined earlier, because of the difficulties in estimating prospect theory parameters from accept−reject task data, we did not compare the λ values from our range manipulation in Experiment 2 with the λ values from Walasek and Stewart. Using the AUC measure, in all four of Walasek and Stewart’s experiments, the effect of range was replicated. Table 2 shows that in every experiment the AUC was larger in the G_Wide_ – L_Narrow_ condition than in the G_Narrow_ – L_Wide_ condition. This is the pattern that we saw here in Experiment 2’s range condition (the only experiment with a range manipulation). In summary, the effects of range, which is what DbS predicts, were robust and well replicated.[Table-anchor tbl2]

The effect of range in Walasek and Stewart’s (2015) data is about half of what DbS predicts, assuming only intraexperiment comparisons, and the effect in the range conditions of Experiment 2 is only about one quarter of the size of the effect that DbS predicts, assuming only intraexperiment comparisons.

A core claim in the original article (Walasek & Stewart, 2015) was that loss aversion could be reversed. In the original λ analysis, they saw reversal and elimination of loss aversion in three out four experiments. In the new AUC reanalysis, the reversal occurred in only one of them (Experiment 1b). One can still see the reversal of loss aversion in one experiment but not in the others. We know of at least one other lab that replicated the reversal of loss aversion using the same paradigm (M. Jung, personal communication, April 17, 2018).

Apart from the central role played by rank in the DbS, DbS’s account of loss aversion rests on the assumption that gains and losses are evaluated separately. Without separate evaluation, there would be no effects of range or skew. It is commonly assumed that when people evaluate positive and negative outcomes or events, they engage mainly in within-domain comparisons (McGraw, Larsen, Kahneman, & Schkade, 2010). But allowing cross-domain comparisons, so that the magnitudes of gains are sometimes compared with losses, and vice versa, would also act to reduce the size of the range and rank effects we measured here (see also Noguchi & Stewart, in press, for an example of cross-dimension comparisons in a DbS account of multiattribute choice). Other accounts posit that the reference group for evaluating different outcomes may incorporate both gains and losses. For example, lotteries tend to be rated as more attractive when they include a low chance of a small loss. For example, the gamble 95% chance of $20 or 5% chance of $0 is made more attractive by swapping the $0 for a tiny −9-cent loss because, according to Slovic, Finucane, Peters, and MacGregor (2007; Yechiam & Hochman, 2013a), people do not spontaneously consider losses when they evaluate a lottery in the gain domain only. However, once losses are involved, the comparison sample can include both gains and losses, which makes the available positive outcomes seem more attractive in comparison. If people engage in across-domain comparisons, one would not observe any context effects when the range and skewness are manipulated. So, as with comparisons to distributions of gains and losses outside the experimental context described earlier, cross-domain comparisons are also a possible account of the smaller size of range and rank effects.

### Conclusion

This work extends the understanding of the processes underlying context effects in elicited loss aversion. We found small but robust effects of the manipulation of the skew of the distributions of gains and losses upon loss aversion. This means that the rank hypothesis embodied in range-frequency theory and decision by sampling is responsible, at least in part, for loss aversion in the accept−reject task. Our findings offer insights on the limits of the context sensitivity of loss aversion (Ert & Erev, 2011).

## Figures and Tables

**Table 1 tbl1:** Median Parameter Estimates Using the Four-Parameter Version of Prospect Theory for Experiments 1–3

Experiment and condition	α [95% CI]	λ [95% CI]	Bias [95% CI]	w(1/2) [95% CI]	AUC [95% CI]
Experiment 1					
G_Neg_ – L_Pos_	.557 [.378, .712]	1.327 [1.126, 1.495]	1.093 [−.819, 2.616]	3.747 [.926, 5.814]	.346 [.295, .399]
G_Uni_ – L_Uni_	.671 [.430, .891]	1.287 [1.123, 1.420]	1.265 [.282, 2.111]	2.877 [1.691, 4.603]	.424 [.389, .467]
G_Pos_ – N_Neg_	1.24 [.677, 1.833]	1.473 [1.187, 1.728]	2.823 [−.128, 4.941]	4.417 [.953, 7.692]	.426 [.373, .475]
Skew diff	.683 [.108, 1.312]	.146 [−.183, .477]	1.73 [−1.550, 4.566]	.671 [−3.221, 5.125]	.08 [.005, .149]
Experiment 2					
G_Neg_ – L_Pos_	.457 [.285, .594]	1.419 [1.204, 1.569]	1.536 [−.000, 3.164]	3.726 [.683, 5.672]	.277 [.245, .310]
G_Pos_ – L_Neg_	.819 [.515, 1.094]	1.556 [1.189, 1.882]	1.365 [.426, 2.374]	2.702 [.475, 4.961]	.391 [.365, .425]
Skew diff	.361 [.036, .694]	.136 [−.245, .542]	−.171 [−2.034, 1.671]	−1.023 [−3.846, 2.927]	.115 [.073, .162]
G_Narrow_ – L_Wide_	1.075 [.584, 1.589]	1.344 [1.064, 1.587]	.465 [.029, .824]	1.715 [1.270, 2.177]	.383 [.307, .431]
G_Wide_ – L_Narrow_	.655 [.527, .805]	1.583 [1.428, 1.735]	1.242 [−.321, 2.529]	2.163 [1.333, 3.316]	.306 [.266, .342]
Range diff	.419 [−.103, .944]	−.239 [−.559, .047]	−.777 [−2.150, .796]	−.448 [−1.686, .485]	.077 [−.007, .137]
Experiment 3					
G_Neg_ – L_Pos_	.501 [.142, .774]	1.302 [1.082, 1.458]	2.224 [.693, 4.356]	14.309 [7.891, 22.572]	.353 [.320, .395]
G_Pos_ – L_Neg_	1.243 [.768, 1.636]	1.391 [.986, 1.784]	2.355 [−.150, 4.042]	7.753 [6.548, 9.624]	.426 [.370, .497]
Skew diff	.742 [.203, 1.284]	.089 [−.326, .557]	.13 [−3.348, 2.191]	−6.556 [−14.664, .374]	.074 [.002, .149]
*Note.* AUC = area under the curve; G = gain; L = loss; Neg = negative; Pos = positive; Uni = uniform; Diff = difference.					

**Table 2 tbl2:** Median AUC Estimates in Walasek and Stewart’s (2015) Experiments 1–3 Based on the Four-Parameter Version of Prospect Theory

Experiment and condition	AUC [95% CI]
Experiment 1A	
G_Uni(20)_ – L_Uni(20)_	.467 [.425, .510]
G_Uni(40)_ – L_Uni(40)_	.477 [.454, .515]
G_Narrow(20)_ – L_Wide(40)_	.490 [.436, .525]
G_Wide(40)_ – L_Narrow(20)_	.252 [.223, .283]
Experiment 1B	
G_Wide(40)_ – L_Narrow(20)_	.302 [.269, .336]
G_Narrow(20)_ – L_Wide(40)_	.557 [.526, .598]
G_Uni(20)_ – L_Uni(20)_	.464 [.423, .494]
G_Uni(40)_ – L_Uni(40)_	.483 [.452, .508]
Experiment 2	
G_Wide(60)_ – L_Narrow(20)_	.216 [.183, .242]
G_Narrow(20)_ – L_Wide(60)_	.393 [.361, .422]
Experiment 3	
G_Wide(40)_ – L_Narrow(20)_	.258 [.181, .310]
G_Narrow(20)_ – L_Wide(40)_	.510 [.456, .561]
G_Uni(40)_ – L_Uni(40)_	.316 [.241, .382]
Range difference	
Experiment 1A	.238 [.173, .282]
Experiment 1B	.255 [.210, .309]
Experiment 2	.177 [.137, .222]
Experiment 3	.252 [.180, .347]
*Note.* Subscripted numbers in parentheses indicate the highest gains and losses used in those groups. AUC = area under the curve; CI = confidence interval; G = gain; L = loss; Uni = uniform.	

**Figure C1 fig7:**
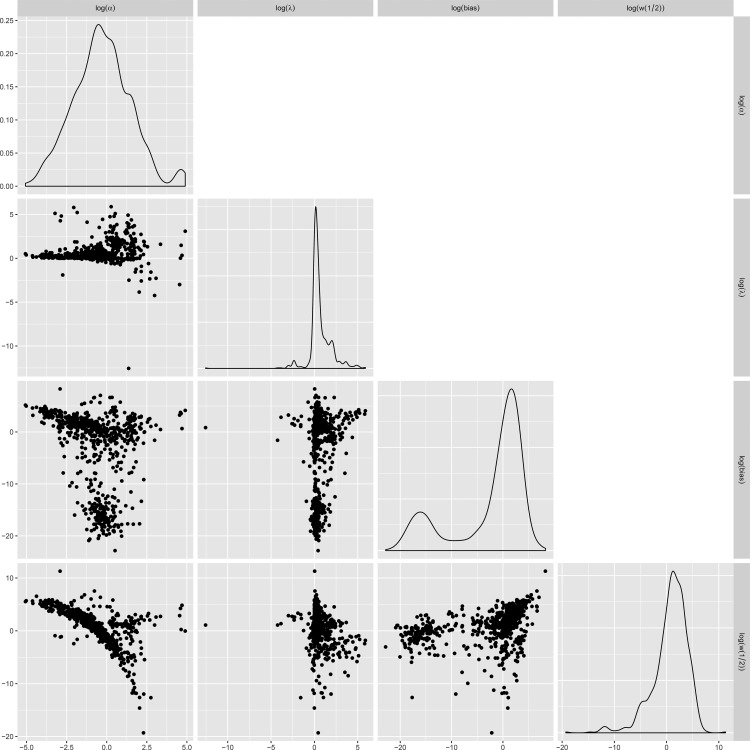
Parameter correlations in Experiment 1.

## A The Distributions of Gains and Losses Used in the Experiments

**Table:**

Distribution and domain	Distribution of outcomes
G_Pos_ – L_Neg_	
Gains	1, 2, 4, 10, 16, 23, 31
Losses	−1, −9, −16, −22, −27, −30, −31
G_Neg_ – L_Pos_	
Gains	1, 9, 16, 22, 27, 30, 31
Losses	−1, −2, −4, −10, −16, −23, −31
G_Uni_ – L_Uni_	
Gains	1, 6, 11, 16, 21, 26, 31
Losses	−1, −6, −11, −16, −21, −26, −31
G_Wide_ – L_Narrow_	
Gains	2, 12, 22, 32, 42, 52, 62
Losses	−1, −6, −11, −16, −21, −26, −31
G_Narrow_ – L_Wide_	
Gains	1, 6, 11, 16, 21, 26, 31
Losses	−2, −12, −22, −32, −42, −52, −62
*Note.* G = gain; L = loss; Pos = positive; Neg = negative; Uni = uniform.	

## B Median Parameter Estimates in Every Condition in Experiments 1, 2, and 3 Using the Five-Parameter Version of the Prospect Theory

**Table:**

Experiment and condition	α [95% CI]	β [95% CI]	λ [95% CI]	bias [95% CI]	w(1/2) [95% CI]	AUC [95% CI]
Experiment 1						
G_Neg_ – L_Pos_	.836 [.674, .959]	.910 [.816, 1.053]	1.486 [1.300, 1.693]	.003 [−.044, .030]	1.004 [.465, 1.367]	.339 [.294, .380]
G_Uni_ – L_Uni_	.868 [.687, 1.033]	.838 [.715, .959]	1.416 [1.216, 1.692]	.067 [−.395, .361]	1.58 [.817, 2.457]	.426 [.387, .488]
G_Pos_ – N_Neg_	1.115 [.643, 1.624]	1.263 [.585, 1.788]	1.272 [.957, 1.573]	.763 [.281, 1.302]	1.436 [.137, 2.388]	.418 [.372, .464]
Skew diff	.280 [−.194, .827]	.353 [−.361, .864]	−.215 [−.595, .132]	.759 [.287, 1.311]	.432 [−.870, 1.563]	.079 [.019, .144]
Experiment 2						
G_Neg_ – L_Pos_	.693 [.564, .793]	.855 [.681, 1.050]	1.489 [1.299, 1.708]	.075 [−.269, .313]	1.530 [1.033, 2.000]	.287 [.252, .321]
G_Pos_ – L_Neg_	.879 [.591, 1.073]	1.056 [.635, 1.393]	1.430 [1.107, 1.807]	.247 [−.162, .524]	1.395 [.693, 1.981]	.390 [.362, .433]
Skew diff	.185 [−.113, .420]	.201 [−.271, .568]	−.059 [−.455, .360]	.172 [−.295, .612]	−.135 [−.986, .627]	.103 [.062, .160]
G_Narrow_ – L_Wide_	.965 [.755, 1.113]	1.220 [.819, 1.565]	1.178 [1.070, 1.327]	.056 [−.033, .145]	1.177 [.647, 1.742]	.397 [.329, .451]
G_Wide_ – L_Narrow_	.728 [.636, .830]	.844 [.745, .950]	1.242 [1.089, 1.412]	.003 [−.025, .023]	.879 [.218, 1.359]	.304 [.251, .356]
Range diff	.237 [−.004, .406]	.376 [−.044, .732]	−.064 [−.257, .153]	.053 [−.035, .149]	.298 [−.387, 1.199]	.093 [.006, .167]
Experiment 3						
G_Neg_ – L_Pos_	.867 [.691, 1.017]	.827 [.562, .992]	1.541 [1.142, 2.108]	.000 [−.024, .018]	1.144 [.332, 1.647]	.343 [.309, .375]
G_Pos_ – L_Neg_	1.273 [.974, 1.469]	1.644 [1.235, 2.131]	1.340 [.881, 1.759]	.179 [−.159, .478]	1.310 [.355, 2.200]	.411 [.345, .474]
Skew diff	.406 [.072, .664]	.817 [.408, 1.404]	−.201 [−.961, .352]	.178 [−.157, .482]	.166 [−.837, 1.414]	.068 [−.005, .140]
*Note.* CI = confidence interval; AUC = area under the curve; G = gain; L = loss; Neg = negative; Pos = positive; Uni = uniform; diff = difference.						

## C Scatterplots for Pairs of Parameters Estimated Using Equation 2 in Experiment 1 Figure C1

[Fig-anchor fig7]

## References

[c1] Camerer C. F. (2005). Three cheers—psychological, theoretical, empirical—for loss aversion. Journal of Marketing Research, 42, 129–133. 10.1509/jmkr.42.2.129.62286

[c2] Chib V. S., De Martino B., Shimojo S., & O’Doherty J. P. (2012). Neural mechanisms underlying paradoxical performance for monetary incentives are driven by loss aversion. Neuron, 74, 582–594. 10.1016/j.neuron.2012.02.038 22578508 PMC3437564

[c3] Chumbley J. R., Krajbich I., Engelmann J. B., Russell E., Van Uum S., Koren G., & Fehr E. (2014). Endogenous cortisol predicts decreased loss aversion in young men. Psychological Science, 25, 2102–2105. 10.1177/0956797614546555 25212582

[c4] De Martino B., Camerer C. F., & Adolphs R. (2010). Amygdala damage eliminates monetary loss aversion. Proceedings of the National Academy of Sciences of the United States of America, 107, 3788–3792. 10.1073/pnas.0910230107 20142490 PMC2840433

[c5] Ert E., & Erev I. (2011). On the descriptive value of loss aversion in decisions under risk. SSRN Electronic Journal, 8, 214–235.

[c6] Frydman C., Camerer C., Bossaerts P., & Rangel A. (2011). MAOA-L carriers are better at making optimal financial decisions under risk. Proceedings of the Royal Society B: Biological Sciences, 278, 2053–2059. 10.1098/rspb.2010.2304 PMC3107654 21147794

[c7] Hertwig R., Barron G., Weber E. U., & Erev I. (2004). Decisions from experience and the effect of rare events in risky choice. Psychological Science, 15, 534–539. 10.1111/j.0956-7976.2004.00715.x 15270998

[c8] Hjorth K., & Fosgerau M. (2011). Loss aversion and individual characteristics. Environmental and Resource Economics, 49, 573–596. 10.1007/s10640-010-9455-5

[c9] Kahneman D., & Tversky A. (1979). Prospect theory: An analysis of decision under risk. Econometrica, 47, 263–292. 10.2307/1914185

[c10] Kőszegi B., & Rabin M. (2006). A model of reference-dependent preferences. Quarterly Journal of Economics, 121, 1133–1165.

[c51] McGraw A. P., Larsen J. T., Kahneman D., & Schkade D. (2010). Comparing gains and losses. Psychological Science, 21, 1438–1445. 10.1177/0956797610381504 20739673

[c11] Nilsson H., Rieskamp J., & Wagenmakers E.-J. (2011). Hierarchical Bayesian parameter estimation for cumulative prospect theory. Journal of Mathematical Psychology, 55, 84–93. 10.1016/j.jmp.2010.08.006

[c12] Noguchi T., & Stewart N. (2014). In the attraction, compromise, and similarity effects, alternatives are repeatedly compared in pairs on single dimensions. Cognition, 132, 44–56. 10.1016/j.cognition.2014.03.006 24762922

[c13] Noguchi T., & Stewart N. (in press). Multialternative decision by sampling. Psychological Review. 10.1037/rev0000102 PMC6022729 29952622

[c14] Pachur T., & Kellen D. (2013). Modeling gain-loss asymmetries in risky choice: The critical role of probability weighting In Knauff M., Pauen M., Sebanz N., & Wachsmuth I. (Eds.), Cooperative minds: Social interaction and group dynamics: Proceedings of the 35th Annual Meeting of the Cognitive Science Society (pp. 3205–3210). Austin, TX: Cognitive Science Society.

[c15] Parducci A. (1965). Category judgment: A range-frequency model. Psychological Review, 72, 407–418. 10.1037/h0022602 5852241

[c16] Slovic P., Finucane M. L., Peters E., & MacGregor D. G. (2007). The affect heuristic. European Journal of Operational Research, 177, 1333–1352. 10.1016/j.ejor.2005.04.006

[c17] Sokol-Hessner P., Camerer C. F., & Phelps E. A. (2013). Emotion regulation reduces loss aversion and decreases amygdala responses to losses. Social Cognitive and Affective Neuroscience, 8, 341–350. 10.1093/scan/nss002 22275168 PMC3594725

[c18] Spektor M. S., & Kellen D. (2018). The relative merit of empirical priors in non-identifiable and sloppy models: Applications to models of learning and decision-making: Empirical priors. Psychonomic Bulletin & Review. Advance online publication 10.3758/s13423-018-1446-5 29589289

[c19] Stewart N., Chater N., & Brown G. D. A. (2006). Decision by sampling. Cognitive Psychology, 53, 1–26. 10.1016/j.cogpsych.2005.10.003 16438947

[c20] Stewart N., Reimers S., & Harris A. J. L. (2015). On the origin of utility, weighting, and discounting functions: How they get their shapes and how to change their shapes. Management Science, 61, 687–705. 10.1287/mnsc.2013.1853

[c21] Stewart N., Scheibehenne B., & Pachur T. (2018). Psychological parameters have units: A bug fix for stochastic prospect theory and other decision models. 10.17605/OSF.IO/QVGCD

[c22] Stewart N., & Simpson K. (2008). A decision-by-sampling account of decision under risk In Chater N. & Oaksford M. (Eds.), The probabilistic mind: Prospects for Bayesian cognitive science (pp. 261–276). Oxford, UK: Oxford University Press 10.1093/acprof:oso/9780199216093.003.0012

[c23] Tanaka T., Camerer C. F., & Nguyen Q. (2010). Risk and time preferences: Experimental and household survey data from Vietnam. American Economic Review, 100, 557–571. 10.1257/aer.100.1.557

[c24] Tanaka Y., & Munro A. (2014). Regional variation in risk and time preferences: Evidence from a large-scale field experiment in rural Uganda. Journal of African Economies, 23, 151–187. 10.1093/jae/ejt020

[c25] Tom S. M., Fox C. R., Trepel C., & Poldrack R. A. (2007, 1 26). The neural basis of loss aversion in decision-making under risk. Science, 315, 515–518. 10.1126/science.1134239 17255512

[c26] Tversky A., & Kahneman D. (1992). Advances in prospect theory: Cumulative representation of uncertainty. Journal of Risk and Uncertainty, 5, 297–323. 10.1007/BF00122574

[c27] Usher M., & McClelland J. L. (2004). Loss aversion and inhibition in dynamical models of multialternative choice. Psychological Review, 111, 757–769. 10.1037/0033-295X.111.3.757 15250782

[c28] Walasek L., & Stewart N. (2015). How to make loss aversion disappear and reverse: Tests of the decision by sampling origin of loss aversion. Journal of Experimental Psychology: General, 144, 7–11. 10.1037/xge0000039 25485606 PMC4312134

[c29] Walasek L., & Stewart N. (2018). You cannot estimate an individual’s loss aversion using an accept−reject task. Manuscript submitted for publication.

[c30] Wolf L. K., Wright N. D., Kilford E. J., Dolan R. J., & Blakemore S. J. (2013). Developmental changes in effects of risk and valence on adolescent decision-making. Cognitive Development, 28, 290–299. 10.1016/j.cogdev.2013.04.001 24027353 PMC3765945

[c31] Yechiam E., & Hochman G. (2013a). Loss-aversion or loss-attention: The impact of losses on cognitive performance. Cognitive Psychology, 66, 212–231. 10.1016/j.cogpsych.2012.12.001 23334108

[c32] Yechiam E., & Hochman G. (2013b). Losses as modulators of attention: Review and analysis of the unique effects of losses over gains. Psychological Bulletin, 139, 497–518. 10.1037/a0029383 22823738

[c33] Zhong S., Chark R., Ebstein R. P., & Chew S. H. (2012). Imaging genetics for utility of risks over gains and losses. NeuroImage, 59, 540–546. 10.1016/j.neuroimage.2011.07.031 21801841

